# No Time to Lift? Designing Time-Efficient Training Programs for Strength and Hypertrophy: A Narrative Review

**DOI:** 10.1007/s40279-021-01490-1

**Published:** 2021-06-14

**Authors:** Vegard M. Iversen, Martin Norum, Brad J. Schoenfeld, Marius S. Fimland

**Affiliations:** 1 Department of Public Health and Nursing, Faculty of Medicine and Health Sciences, Norwegian University of Science and Technology, Trondheim, Norway; 2 Department of Neuromedicine and Movement Science, Faculty of Medicine and Health Sciences, Norwegian University of Science and Technology, Trondheim, Norway; 3 Independent Researcher, Norum Helse AS, Oslo, Norway; 4 Department of Health Sciences, CUNY Lehman College, Bronx, NY USA; 5 Unicare Helsefort Rehabilitation Centre, Rissa, Norway

## Abstract

**Abstract:**

Lack of time is among the more commonly reported barriers for abstention from exercise programs. The aim of this review was to determine how strength training can be most effectively carried out in a time-efficient manner by critically evaluating research on acute training variables, advanced training techniques, and the need for warm-up and stretching. When programming strength training for optimum time-efficiency we recommend prioritizing bilateral, multi-joint exercises that include full dynamic movements (i.e. both eccentric and concentric muscle actions), and to perform a minimum of one leg pressing exercise (e.g. squats), one upper-body pulling exercise (e.g. pull-up) and one upper-body pushing exercise (e.g. bench press). Exercises can be performed with machines and/or free weights based on training goals, availability, and personal preferences. Weekly training volume is more important than training frequency and we recommend performing a minimum of 4 weekly sets per muscle group using a 6–15 RM loading range (15–40 repetitions can be used if training is performed to volitional failure). Advanced training techniques, such as supersets, drop sets and rest-pause training roughly halves training time compared to traditional training, while maintaining training volume. However, these methods are probably better at inducing hypertrophy than muscular strength, and more research is needed on longitudinal training effects. Finally, we advise restricting the warm-up to exercise-specific warm-ups, and only prioritize stretching if the goal of training is to increase flexibility. This review shows how acute training variables can be manipulated, and how specific training techniques can be used to optimize the training response: time ratio in regard to improvements in strength and hypertrophy.

**Graphic Abstract:**

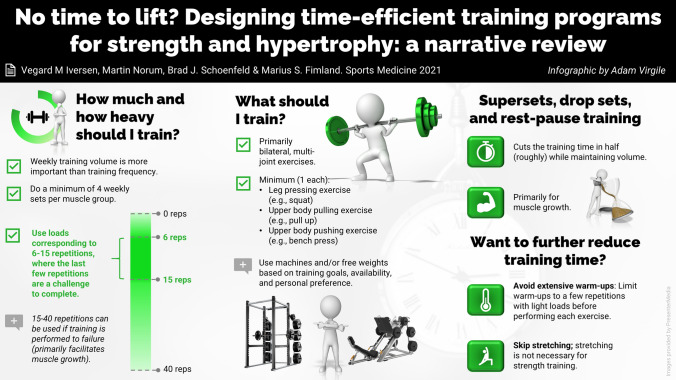

## Key Points

**Table:** 

Strength training is good for health, but lack of time is a barrier for many individuals.
Strength training can be made more time-efficient by prioritizing bilateral, multijoint movements through a full range of motion with ≥ 4 weekly sets per muscle group using a 6–15 RM loading range.
Supersets, drop sets and rest-pause training roughly halves training time compared to traditional training, while maintaining training volume.
Restrict the warm-up to exercise-specific warm-ups.
Only prioritize stretching if the goal of training is to increase flexibility.

## Introduction

Strength training increases muscular strength and hypertrophy, and provides numerous other positive health benefits, including improved functional ability, cardio-metabolic risk profile and well-being [1, 2]. Strength training is therefore recommended as an interventional strategy for the general population [1, 3]. However, a majority of people refrain from performing strength training and other forms of exercise, and as much as a quarter of the world’s population are at risk for developing health-related problems and diseases linked to inactivity [4]. Therefore, it is necessary to find ways to engage more people in both daily-life physical activities and regimented forms of exercise such as strength training, which is one of the most popular forms of exercise globally [5]. Lack of time is a common self-reported barrier to engagement in structured exercise [6, 7]. Thus, understanding how strength training programs can be designed in ways that reduce training time without meaningfully compromising results could encourage more people to engage in this form of exercise.

A typical strength and hypertrophy program for untrained or intermediately trained healthy adults involves training all major muscle groups with 2–4 sets of 8–10 exercises for 3–12 repetitions with 2–5 min rest between sets, carried out 2–4 times per week [1, 8]. Including a warm-up and stretching, traditional strength training programs often exceed an hour in length over several sessions per week. The aim of this narrative review is to synthesize the evidence as to how strength training can be programmed for optimum time-efficiency. Our primary focus is upon manipulation of training variables—i.e. frequency, volume, load, exercise selection, muscle action, repetition velocity and rest periods [9]. We also cover the efficacy of several popular time-saving advanced training techniques (i.e. supersets-, dropsets-, and rest-pause training), whether warm-ups and stretching should be included, and how much training is required to maintain strength and muscle mass. We emphasize that this narrative review is intended for those in the general public that have limited time for training, and not for those who are seeking to optimize training adaptations without regard to a time commitment (e.g. athletes). While these time saving methods are relevant to everyone looking to limit training time, most studies referred to in this review included samples of untrained or recreationally active individuals. Where applicable, we have specified when resistance-trained individuals were included in the study samples.

## Training Frequency and Volume

Training frequency and training volume are arguably the most important variables related to training time. General guidelines recommend that people train 2–3 times per week [1]; unfortunately, this recommendation may cause those who find it challenging to train several times a week to not train at all. However, emerging evidence indicates that it is possible to achieve similar training effects by training once a week compared to a higher frequency when total weekly volume is equated [10, 11]. In a meta-analysis from 2018, Ralston et al. compared strength gains from low training frequency (1 day per week), medium training frequency (2 days per week), and high training frequency (≥ 3 days per week) for each muscle group [10]. The authors reported only negligibly greater increases in strength gains from higher frequencies for a mixed population. Also, when training volume was matched, i.e. total number of repetitions (sets × repetitions) or as total volume loading (sets × repetitions × loads), no significant effect of training frequency was observed for strength gains. Thus, training a muscle 1 day per week appears to induce similar strength gains as training ≥ 3 times per week if the total training volume is the same. Still, in real-life situations, a higher training frequency allows for a higher training volume and therefore often results in greater strength gains as demonstrated in a meta-analysis by Grgic et al. [12]. A recently published meta-analysis by Schoenfeld et al. found no compelling evidence that training frequency confers a meaningful impact on muscle hypertrophy when training volume is matched [11]. However, as higher training volumes can be expected from higher training frequencies in real-life situations, a higher training frequency is likely preferable for those seeking to maximize muscle strength and hypertrophy regardless of the time commitment. Alternatively, for those seeking to minimize training time, it appears more important to focus on acquiring a sufficient weekly training volume than to focus on a given training frequency.

So-called “micro dosing”, i.e. frequent training sessions of very short duration (e.g. 15 min), could be a viable alternative to traditional programs. There are few studies and results should be interpreted with caution, but in line with the notion that the total weekly volume is the primary determinant of gains in muscle mass and strength, they generally show similar adaptations to traditional programs [13, 14]. Thus, very short and frequent workout sessions can be a viable alternative for individuals reluctant to schedule longer training sessions.

Regarding weekly training volume, current guidelines recommend performing 2–4 sets per muscle group for 2–3 times a week [1], which corresponds to a weekly training volume of 4–12 sets per muscle group. Thus, there is a wide gap in recommended sets, and while higher training volumes may be more beneficial for gains in strength and muscle mass [15], evidence shows that significant muscular gains can be obtained from a low training volume as well [16–18]. Several studies have demonstrated that performing only a single set three times per week is effective for increasing strength and hypertrophy [16, 17], and the American College of Sports Medicine (ACSM) states that performing a single set 2–3 times per week can be beneficial especially for older individuals and novice trainees. The results from a recent meta-analysis by Androulakis–Korakis et al. indicated that single-set training also can have a positive impact on trained individuals [18]. Although the effect was suboptimal, performing a single set of 6–12 repetitions, using 70–85% of 1 RM loading, for two to three times per week was identified as the minimum effective training dose to increase 1 RM strength in resistance-trained men (defined as having a minimum of one year of resistance training experience) [18].

It also is possible that different muscle groups require different stimuli. Ronnestad et al. [19] demonstrated that untrained individuals achieved similar improvements in strength and hypertrophy in upper-body muscles from training with one vs. three sets over three times per week while three sets per session were superior for improvements in the leg muscles. This is consistent with a 2019 meta-analysis of resistance training for astronauts that demonstrated performing a single-set per session resulted in similar strength improvements in upper-body exercises as three-sets per session, while three sets per muscle were superior for muscles in the lower body [20]. Thus, when time is of the essence, untrained individuals should consider performing more weekly sets for the lower body musculature and restrict time spent on upper-body training. However, the required stimuli for upper-body muscles increases when people become more experienced, and trained individuals appear to achieve superior adaptations from three vs one set of training for both the upper and lower body muscles [20]. Thus, the trade-off must be considered between time-efficiency and maximizing gains.

Some studies have tried to differentiate and quantify the effects on strength and hypertrophy of varying numbers of training sets [21–23]. Early meta-analytic data from Krieger reported that the magnitude of gains in strength and hypertrophy are respectively 46% and 40% higher when performing multiple sets per exercise per training session compared to single sets [21, 22]. However, a moderate effect was shown for performing single sets as well, with effect sizes of 0.54 and 0.25 for strength and hypertrophy, respectively. Importantly, the effect of increasing from 1 set to 2–3 sets was greater than increasing from 2–3 to 4–6 sets. La Scala et al. also found that performing a relatively low number of sets (< 3) twice per week significantly increased upper-body muscle mass, and that performing additional sets only provided small incremental benefits [24]. However, the optimal number of sets is still a controversial topic, and some authors advocate the necessity of high volume (> 10 weekly sets) to optimize the hypertrophic response [15]. A 2017 meta-analysis by Schoenfeld et al. stratified hypertrophic gains across the pooled literature for < 5 weekly sets, 5–9 weekly sets, and 10 + weekly sets, reporting increases of ~ 5%, ~ 7%, and ~ 10%, respectively [23]. These findings indicate that although a high training volume appears superior to maximize muscular adaptations, it is possible to improve both strength and hypertrophy when training with a relatively low number of weekly sets (< 5 sets). However, < 5 weekly sets can refer to anything from 1 to 4 sets and considering that there still is a lack of consensus regarding this metric, we advise to perform at least 4 weekly sets per muscle; the inclusion of higher training volumes should be determined based on individual response, taking into account whether the additional time expenditure is worth the potential additive increases in muscular adaptations. These sets can be distributed throughout the week as desired. This has important implications for those who are time-pressed as lower volume routines represent a viable option to balance efficiency with results.

## Training Load and Repetitions

A 2005 review by Bird et al. suggested that training load—usually defined as target repetition number to muscular failure (e.g. 12 RM) or as a percentage of the one repetition maximum (% of 1 RM)—is the most important variable in strength training [9]. A traditional belief has been that adaptations following strength training are load dependent, with heavy loads, moderate loads and low loads used for increasing maximum strength, hypertrophy and muscular endurance, respectively [9]. The ACSM guidelines recommend people in general train within a 1–12 RM loading range with emphasis on the 6–12 RM range to improve muscle strength and hypertrophy, with lighter loads (15–25 RM) suggested for increasing muscular endurance [1, 8]. However, emerging evidence indicates that similar hypertrophic responses occur across a wide spectrum of repetition ranges (even when using very light weights) as long as the training is performed with a high level of effort and the number of sets is equated [25]. In their 2017 meta-analysis of 21 studies, Schoenfeld et al. investigated the effects of training with high loads (i.e. ≥ 60% of 1 RM or ≤ 15 RM) compared to low-loads (i.e. < 60% of 1 RM or > 15 RM with most studies utilizing a 15–40 repetitions range) and found similar increases in hypertrophy, irrespective of the magnitude of load. Furthermore, although the use of heavy-loads was superior for inducing strength gains, considerable strength increases were shown for low-load training as well (increases in 1RM of 35% and 28%, respectively) [25]. Most studies on the topic involved untrained individuals; that said, similar results have been found in resistance-trained individuals with respect to muscle hypertrophy, but heavy loads appear to be more important for strength gains in this population [26, 27].

With respect to time-efficiency it can be argued that heavy-load training is preferable as fewer repetitions means less training time. Performing a high number of repetitions is also metabolically taxing, and accordingly a higher perceived discomfort has been reported with high-repetition training (using 50% of 1RM) compared to low repetition training (using 80% of 1RM) [28]. It also is worth mentioning that regularly training to muscular failure is not essential to increase muscular growth and strength gains when heavy-loads are used [29]. Thus, heavier loads may be preferable when training time is limited, and it seems reasonable to emphasize the 6–12 RM range as recommended by the ACSM as a vast body of evidence indicates that this loading zone is very effective for increasing maximal strength and hypertrophy. However, low-load training provides a time-efficient alternative for home-based training, discussed in Sect. 9, and also represents a viable alternative to heavy-load training for those with joint-related issues (e.g. osteoarthritis, etc.).

## Exercise Selection

### Multi-Joint and Single Joint Exercises

There are a myriad of exercises to choose from when designing a strength training program. On a basic level, strength training exercises can be divided into single-joint exercises (or isolation exercises) and multi-joint exercises (or compound exercises). Single-joint exercises are designed to target specific muscles; examples include the biceps curl, shoulder abduction, and leg extension. Alternatively, multi-joint exercises activate several groups of muscles synchronously, which allows lifting of heavier weights; examples include the squat, bench press and barbell row. ACSM guidelines state that the strength training programs should include both single- and multi-joint exercises, but recommend emphasizing multi-joint exercises as they are considered more effective in increasing overall strength and daily-life function [1]. Some studies have suggested that hypertrophy occurs earlier following single-joint exercises as these exercises generally are easier to learn and thus require less neural adaptation than multi-joint exercises [30, 31]. However, strength improvements in multi-joint exercises appear to be higher and more rapid than in single-joint exercises [32]. Thus, single-joint exercises could provide little added benefit from a strength standpoint. A review from 2017 that encompassed 23 original articles concluded that, at least for upper-body training, it appears unlikely that the inclusion of single-joint exercises will meaningfully contribute to additional short- or long-term benefits over training solely with multi-joint exercises [33].

The role of single-joint exercises remains equivocal and further research is needed to better understand their impact on long-term hypertrophic responses, whether response varies between muscles (even portions of the muscles) and individuals with different training status, and the extent to which they provide functional and/or sport-specific enhancements [34, 35]. Despite the current gaps in the literature, it seems unlikely that the use of single joint exercises would provide substantial additional training benefits for the general public compared to training only with multi-joint exercises, especially for individuals with limited training experience. Thus, for those seeking time-efficiency in their workouts, we recommend prioritizing multi-joint exercises as the greater amount of muscle mass trained allows for shorter training sessions, despite the somewhat longer recovery needed between sets to accommodate the higher levels of exertion.

### Free-Weight and Machine Exercises

External loading in resistance training can be provided by a variety of different exercise equipment, with free-weights (i.e. barbells and dumbbells) and strength training machines being among the most popular. Both modalities can be used effectively to increase strength and hypertrophy, and there is no strong scientific evidence indicating either of the modalities being superior to the other [36]. The main difference between modalities is that it is easier to simulate real-life movements and sport-specific movements with free-weights compared to most machines, which usually have limited adaptability of the movement pattern. However, the variety of machines is vast, with some allowing for training in a manner very similar to free-weights.

Free-weights are very versatile, allowing for a great variety of multi-joint exercises, which again can facilitate time-efficient training sessions. Additionally, free-weights can be used regardless of body-type while machines may not be well-suited to certain body-types. However, free-weights can be more intimidating for novice users than machines [37]. Both modalities are considered safe if proper technique is used, but machine exercises are often perceived as safer than free-weight exercises, which require more knowledge of proper technique and sometimes may necessitate a spotter. However, while free-weight training is associated with higher reported injury rates, most of these injuries are related to weights falling on people and not the modality per se [38]. Still, training with machines facilitates the use of very heavy loads and training to muscular failure without the need for a spotter, which may be especially beneficial for inexperienced lifters.

Free-weight exercises can be performed using a barbell (e.g. bench press) or with dumbbells (e.g. dumbbell press), with both modalities proving effective for stimulating strength and hypertrophy. However, due to lower stability requirements, heavier weights can be lifted with a barbell than with dumbbells [39, 40]. In a cross-over study by Saeterbakken et al. [40], resistance-trained participants were able to perform a 1RM lift with approximately 20% heavier loads during the barbell bench press compared to the dumbbell bench press. During the 1RM lift, comparable pectoralis and deltoideus activation was observed, but synergistic activation of the triceps brachii was considerably higher during the barbell bench press than with dumbbells. The lower triceps activation during dumbbell presses are likely due to the dumbbells not being connected to each other, and thus the triceps have a reduced capacity to actively contribute to the pushing movement. This was also shown for barbell vs. dumbbell shoulder presses [39]. When synthesizing the body of literature, training with a barbell allows for a higher total muscle activation and an ability to lift heavier weights compared to dumbbells. While dumbbell exercises can be good for targeting specific muscles, and provide a freer range of motion which in some cases can be desirable, it would seem that training with a barbell is the more time-efficient option. In our opinion, the decision as to whether barbells should be prioritized over machines would need to take several factors into account (e.g. available equipment, lifting experience or the availability of competent instructors).

### Bilateral and Unilateral Exercises

Strength training exercises can be performed unilaterally (training one side of the body at the time, e.g. split squat or dumbbell curl) or bilaterally (training both sides of the body at the same time, e.g. squat or barbell curl). Due to higher stability, and more total muscle mass involved, training can be performed with heavier weights and higher force-output during many bilateral exercises [41]. However, this would not be an issue during relatively simple exercises such as the arm-curl or machine leg extension. During such simple exercises, some studies have in fact reported a bilateral deficit, operationally defined as an inability of the neuromuscular system to produce maximal force during simultaneous limb movements compared to the force developed when the limbs function separately. However, this effect is not observed in individuals habituated to bilateral training, where in fact bilateral facilitation has been observed [42]. Comparable increases in strength, power and hypertrophy following both unilateral and bilateral training have been demonstrated for both trained [43] and untrained individuals [41, 43, 44]. The ACSM, as well as an updated review from 2018 by Suchomel et al., recommends performing both variations, but emphasizing bilateral exercises [1, 41]. Some authors have noted that unilateral exercises provide the benefit of higher core-activation due to greater stability requirements [39, 45]. It should be noted that there is limited evidence on the difference between unilateral and bilateral training. Considering the current evidence, we propose that bilateral exercises are more time-efficient (since both sides of the body are trained simultaneously) and thus should be prioritized unless core-activation is central to a person’s training goal. That said, unilateral training is a viable option to increase the difficulty of an exercise in situations where less weight is available, such as during home-based training.

### Elastic Resistance Bands

Elastic resistance bands can be a time-efficient alternative when traditional training equipment is not available. Resistance bands are versatile, relatively inexpensive, and require very little space, which makes them useful for home-based training and during travels. Several studies have demonstrated that when resistance is matched (i.e. both groups training with for instance 8 RM loading), training with resistance bands produces similar muscle activation to free-weights and machines during performance of single-joint exercises [46, 47]. Some studies also suggest resistance bands may provide a viable alternative to multi-joint exercises [48–50], although traditional equipment should be preferred, if available, for exercises where very heavy loads can be lifted [48]. A 2019 review by Lopes et al. concluded that for individuals with previous strength training experience, resistance training with elastic bands provides similar strength gains as training with traditional equipment for both upper- and lower body muscles [51]. However, the review only identified 8 longitudinal studies on the topic—three of which included participants with coronary disease or chronic obstructive pulmonary disease—and did not discriminate between single- and multi-joint exercises; in fact, only two of the studies compared strength gains in multi-joint exercises. In one of the studies, Colado et al. found similar improvements in maximal isometric squat-, row-, and back extension strength for physically fit females when training with elastic bands versus traditional equipment following an 8-week full body training program [52]. In the other study, Lubans et al. observed comparable improvements in leg press strength for adolescent boys and girls following eight weeks of full body training using free-weights or elastic bands [53]. Contrarily, Iversen et al. found that leg-muscle activation was significantly lower during squats when training with elastic bands alone compared to using free-weights in a cohort of 30 healthy young men and women with mixed training experience [48]. Thus, we recommend the use of conventional equipment when available for performing heavy multiple-joint exercises for the lower body; otherwise, resistance bands can be a viable training option.

### Bodyweight Training

Body weight training provides a time-efficient alternative to traditional resistance exercise, as this form of training can be performed almost anywhere at any time. Although research has repeatedly demonstrated beneficial effects of bodyweight training for health and cardiovascular function [54, 55], the evidence supporting it as an effective modality for stimulating muscular strength and hypertrophy remains much scarcer than for lifting weights. There is compelling evidence that a small number of upper-body bodyweight exercises can be effective strength training alternatives, such as the pull-up/chin-up [56] and push-up [57]. However, little research has been carried out on bodyweight exercise for the lower limbs.

In theory, bodyweight training could be effective for gaining strength and muscle mass, as these adaptations are obtained by progressively overloading the neuromuscular system irrespective of the type of external resistance. However, bodyweight training presents some practical challenges with respect to altering acute training variables. When using external weights, it is easy to incrementally increase resistance, whereas bodyweight resistance usually requires changing the initial form of the exercise to achieve greater resistances (e.g. changing from push-ups on the knees to push-ups on the toes). Thus, one variation of the exercise may be too easy, while the other may be too difficult. Increasing repetitions is therefore generally required to alter the training stimulus until the individual is strong enough to change the form of the exercise progression. Bodyweight training also requires more knowledge about training to progress by changing the biomechanics of an exercise rather than simply adding more weight. As previously mentioned, if training is performed to muscular failure, using a low load-high repetition approach can be effective for strength and especially hypertrophy. Therefore, a well-planned bodyweight program conceivably could be an effective strategy to improve muscular adaptations.

## Other Variables to Consider

### Muscle Action

Muscle actions can be categorized into concentric (shortening of the muscle), eccentric (lengthening of the muscle), and isometric (no change in muscle length). There are some advantages of isolating each of the muscle actions, such as the ability to exert higher power in eccentric movements and potentially elicit greater hypertrophic adaptations; the ability to work with higher rates of force development in concentric movements; and applying force in pain-free joint angles in rehabilitation settings and focusing on weak points at specific joint angles through isometric movements [58, 59]. However, most strength training exercises, and human motion in general, consist of coupling of concentric and eccentric muscle actions, and optimal training responses rely on training both [60]. Thus, manipulation of muscle action in strength training can be useful in given situations, but generally, dynamic muscle actions coupling concentric-eccentric movements should be employed for time-efficiency [60].

### Repetition Velocity

Repetition velocity (or repetition tempo) is operationally defined as the time it takes to perform the concentric and eccentric muscle actions. The ACSM recommends novices and intermediately trained lifters utilize relatively slow (2 s concentric: 4 s eccentric) to moderate (1–2 s: 1–2 s) repetition velocities, while differing velocities are recommended for experienced lifters. It has been suggested that increasing time under tension by utilizing very slow (10 s: 4 s) movements can result in higher hypertrophic responses when training with submaximal loadings [61], and several popular science articles and internet forums advocate training with increased time under tension for hypertrophy. One of the proposed rationales for slow velocity training is that it may increase time under tension and stimulate muscle growth even when using low loadings. However, a 2015 meta-analysis found that when training is performed to failure, it is unlikely that one particular repetition velocity will result in greater hypertrophic gains than another, as it was found that repetition durations (combined concentric and eccentric) ranging from 0.5 to 8 s resulted in similar muscle growth [62]. Regarding time-efficiency, this information is of interest for people who train at home and do not have heavy weights at their disposal, as using a somewhat slower velocity with lighter loads seemingly can be a viable alternative to increasing number of repetitions. However, there appears to be a threshold for velocity, and utilizing super-slow velocities (≥ 10 s) may actually result in an inferior hypertrophic response compared to using faster velocities, likely due to suboptimal muscle fiber stimulation [62]. It should also be noted that there may be unique differences in the hypertrophic response between muscle-groups, and some muscles may benefit more from faster velocities while other muscles may benefit more from more moderate-to-slower velocities [63].

For strength gains, it appears that both fast (< 1 s:1 s) and moderate-slow (> 1 s:1 s) velocities are effective across different loading ranges, but that fast velocities may be somewhat more effective when training with moderate loads (60–79% of 1RM) [64]. In contrast to when training is performed at or close to failure, some studies suggest that training velocity is more essential when training is not performed to failure. González–Badillo et al. [65] examined the effects of training with maximal intended velocity compared to half-maximal velocity in the bench press for students with strength training experience. After 6 weeks of training—using loads corresponding to 60–80% of 1RM and performing 2–8 repetitions (i.e. training was not taken to failure)—maximal strength (1RM) in the bench press had increased by 18.2% and 9.7% in the maximal intended and half-maximal velocity groups, respectively. In another study, Padulo et al. [66] randomized 20 resistance-trained subjects divided to perform bench press with either a fixed pushing velocity (80–100% of maximal intended velocity) or a self-selected velocity. Both groups trained twice per week for 3 weeks at 85% of 1RM. The group performing repetitions with a fast velocity stopped each set when the velocity dropped > 20% and did not perform more sets when the velocity of the first repetition of a new set fell > 20%, while the other group continued until failure (i.e. each set was performed to muscular failure, and training was ended when participants were unable to perform any more repetitions). Despite performing 62% fewer repetitions, the maximal velocity group improved 1RM-strength by 10.2% compared to < 1% in the self-selected velocity group.

In summary, a wide range of repetition velocities can be utilized to induce muscular adaptations, and manipulation of this variable is unlikely to markedly influence changes in muscle growth. As a general rule, a somewhat faster repetition cadence should be employed when time is of the essence since faster velocities tend to be more time-efficient than slower velocities. Moreover, volitional fast velocities may be preferable for improving strength and power, and super-slow velocities (≥ 10 s) should generally be avoided for either strength, power or hypertrophy.

### Rest Periods

The need to rest between sets is often a frustrating requirement for people with limited time to train. However, adequate rest between sets is considered a crucial program variable for optimizing gains in strength and hypertrophy. The interset rest period allows the body to remove lactic acid, and replenish adenosine triphosphate and phosphocreatine—i.e. organic chemicals important for muscular contraction. Insufficient rest can result in a reduced capacity to maintain high muscular force throughout multiple sets and a lowered training volume load (load × repetitions × sets), which is considered important for improving both hypertrophy and strength [67]. Common guidelines recommend a 3–5 min rest interval when training to maximize strength, a 1–2 min rest interval when the goal is hypertrophy, and a 30–60 s rest interval when the goal is muscular endurance [1, 9]. This advice is reflected in recent reviews, where it was concluded that rest intervals should vary from 2–5 min depending on whether the goal is hypertrophy or strength and power [41]. However, a 2017 systematic review by Grgic et al. encompassing 23 RCTs found evidence that short rest intervals (< 1 min) produced robust strength gains in both untrained and trained individuals (10 of the studies included trained populations) although less so than longer rest intervals [67]. The review also found that 1–2 min rest is sufficient for maximizing muscular strength gains in untrained individuals. It also is interesting to note that some studies have found that trained individuals can build up tolerance for short rest intervals. Two RCTs utilizing 8 weeks of training with 8–12 RM suggested that, compared to using a constant rest period of 2 min, training with a progressively decreasing rest time (2 min reduced to 30 s) produced a similar increase in 1RM strength in both the bench press and squat, as well as isokinetic peak torque in the knee extensors and flexors [68, 69]. However, more research is needed to make clear recommendations regarding building tolerance for short rest intervals. Based on most evidence, we advise untrained individuals to schedule 1–2 min rest intervals and trained individuals ≥ 2 min rest intervals. In the following section, we present training techniques that provide the ability to limit the amount of passive rest without significantly compromising results.

## Advanced Time-Saving Training Methods

### Superset Training

**Fig. 1:**
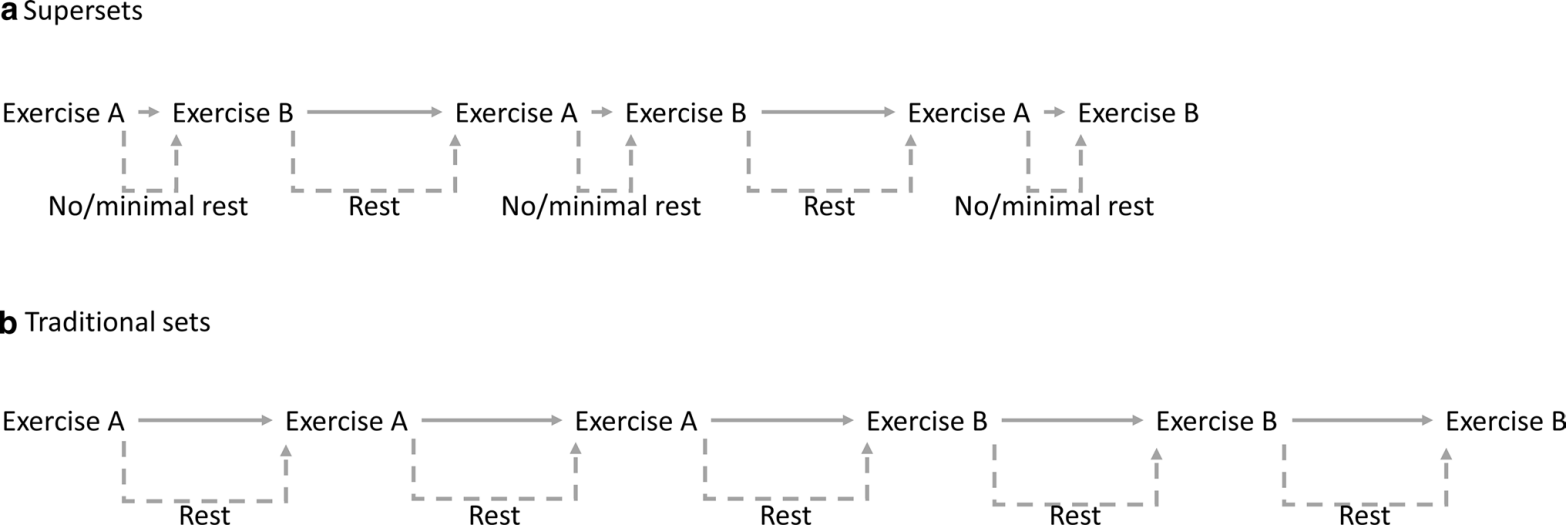
Example of rest intervals in **a** superset, and **b** traditional sets

Superset training has grown in popularity despite limited supporting scientific evidence on the topic. Superset training (also known as paired-sets training or compound sets) refers to the performance of two or more exercises in succession with limited or no rest between them [70] (see Fig. 1 for an example). Since this method substantially limits the time spent at rest, it allows for a greater training density (i.e. performing more exercise in a shorter amount of time) compared to traditional strength training. Supersets can be performed by pairing exercises for the same muscle group (e.g. bench press and flies), or by pairing exercises for different muscle groups (e.g. biceps curl and triceps push-down). Supersetting exercises for the same muscle is primarily a bodybuilding approach where more time can be spent on working individual muscles and hence is generally not relevant from a time-efficiency standpoint. Thus, we will focus on superset training for different muscle groups.

A 2010 review suggested supersetting exercises for agonist and antagonist muscles is a time-efficient alternative to traditional strength training [71]. However, these conclusions can be considered speculative due to the paucity of scientific evidence on the topic at the time. To date, only one longitudinal study has been carried out on superset training using traditional training modalities. In this RCT, Robbins et al. included 15 trained males who performed high-intensity loaded (i.e. 3–6 repetitions) bench press and bench pulls for eight weeks in a superset versus traditional manner [72]. Both groups showed similar improvements in 1-RM bench pull (superset: 2.2 ± 1.1% vs traditional: 1.2 ± 1.7%) and bench press (superset: 2.4 ± 1.8% vs traditional: 2.3 ± 1.9%), bench press throw height using 40% of 1RM (superset: 5.0 ± 16.5% vs traditional: 10.8 ± 10.7%), bench press peak velocity (superset: 4.2 ± 6.3% vs traditional: 3.0 ± 4.0%), and bench press peak power (superset: 9.7 ± 9.2% vs traditional: 9.4 ± 5.4%). However, training time for the superset group was roughly half that for the traditional training group.

Acute cross-over studies support the notion that, when training to failure at an 8-12RM loading scheme, superset training can be performed in approximately half the time as traditional training without compromising training volume [73–76]. All these studies involved supersets that paired exercises for agonists and antagonist muscles. It has been suggested that antagonist preloading potentially can facilitate increased neural activation, which acutely increases strength performance and thereby allows for a higher training volume [74, 75]. Findings from cross-over studies also indicate that superset training induces higher lactate production and higher levels of fatigue than traditional strength training [73, 75–79]. This can substantially reduce the training time, but at the same time decrease neuromuscular performance and force generating capacity during training, particularly for strenuous multi-joint exercises. This hypothesis lacks experimental verification and further research is warranted to better understand the phenomenon.

### Drop Set Training

In drop-set training, training time is reduced by minimizing rest between sets. The strategy involves performing a traditional set, reducing the load, and then immediately performing another set (or multiple sets). Typically, 1–3 drops are used with a 20–25% reduction in weight, with all sets performed to muscular failure [80]. A proposed rationale behind this method is that drop-sets elicit a larger metabolic stress and potentially heightened muscle damage, which in turn could increase the hypertrophic response [80, 81].

Longitudinal studies comparing drop set training to traditional training have generally been unable to detect differences in hypertrophic responses from the strategy [82, 83], but the evidence is both limited and somewhat conflicting. One RCT by Fink et al. suggested that drop set training may be superior for hypertrophy, but inferior for strength [81]. In this study, 16 men (20–23 years with less than one year's participation in regular strength training) engaged in 6 weeks of triceps-push-down training using either drop-set or traditional sets. The traditional group performed three sets to failure at a 12RM load, while the drop-set group performed one set with an initial 12RM load and then reduced the load 20% each time failure was reached for three times with no rest between drops. The cross-sectional area of the triceps increased by 10 ± 4% and 5 ± 2% favoring the drop-set group (not statistically different), while 12RM in triceps push-down increased by 16 ± 12% vs 25 ± 18% favoring the traditional group (not statistically different). While the study sample was small and thus vulnerable to type II errors, the findings indicate that drop-set training can induce increases in both strength and hypertrophy in about half the training time of a traditional training protocol. The average training volume per session (repetitions x load) was similar for the two groups. In another RCT that employed a within-subject design, Ozaki et al. randomized the arms of 9 untrained men to: (1) one set of dumbbell curls (using 80% of 1RM load) followed by four drop-sets using 65%, 50%, 40% and 30% of 1RM, or (2) three “traditional” sets using 80% of 1RM loading, or 3) three sets of “traditional” sets using 30% of 1RM loading [82]. All sets were performed to repetition failure and training was performed 2–3 days per week for 8 weeks. Total training time per session was significantly shorter for the drop-set protocol (~ 2 min) compared to the high-load (~ 7 min) and the low-load protocol (~ 11–12 min). Similar increases in muscle cross-sectional area of the biceps brachii and the brachialis muscles were observed across all groups. Both the high-load and the drop-set protocols increased 1RM strength, but the gains were somewhat higher for the high-load group (not statistically significant—likely due to the study being underpowered).

Despite the limited evidence, drop-set training seems to allow for shorter duration workouts with little or no reductions in training volume or training responses (especially hypertrophy), thus making it a viable training method for those who are time-pressed to train. It should also be noted that most of the studies on the topic were carried out using single joint, upper-body exercises. A recent review stated that while drop sets can be used for both single-joint and multi-joint exercises, the strategy is most suited to single-joint training from a practical perspective [80]. Due to safety concerns, it might not be advisable to include drop-sets in certain compound, free-weight exercises such as squats.

### Rest-Pause Training

The rest-pause method is a method of structuring sets where normal interset rest periods are accompanied by preplanned rest within the training sets [84]. During rest-pause training, sets are segmented into smaller sets with short breaks in between, which are commonly performed in one of two ways. The first approach involves performing 4–6 sets of single repetitions using a load close to 1RM, while the second approach involves performing one set to failure interset rest (often 20 s), new set to failure interset rest, etc., until the preplanned number of repetitions are performed [84].

While reductions in load are necessary during drop-sets, the rationale behind rest-pause training is that the short breaks allow for maintenance of high loads, high concentric velocities, and high power outputs. Thus, rest-pause training conceivably could be a time-efficient strategy for stimulating both muscular strength and hypertrophy (especially the second approach). The acute training effects from rest-pause training have been investigated by Marshal et al., who instructed 14 resistance-trained men to perform 20 repetitions of the back squat using 80% of 1 RM, in three different conditions: (1) 5 × 4 repetitions with 3 min rest and a protocol duration of 780 s; (2) 5 × 4 repetitions with 20 s rest and a protocol duration of 140 s, and; 3) rest-pause: one set to failure with subsequent sets performed to failure with 20 s interset rest and a protocol duration of 103 s [85]. All groups demonstrated comparable decreases in rate of force development immediately after protocol completion with full recovery 5 min after protocol completion, despite a higher muscular activation during the rest-pause condition. The findings strengthen the theory that rest-pause training helps to maintain high concentric force throughout a series of repetitions.

In the only longitudinal study on the topic, Prestes et al. found similar strength gains from rest-pause training and traditional strength training with heavy loading and few repetitions [86]. In this study, 18 trained men performed a two-split training program (i.e. two weekly sessions targeting chest-, shoulder- and arm extensor muscles, and two weekly sessions targeting the leg-, back and arm flexor muscles), including both multi-joint and single-joint exercises. One group performed the exercises in a traditional manner (3 sets of 6 repetitions with 80% of 1 RM loading, with 2–3 min rest between sets) while the other group performed the exercises in a rest-pause manner (i.e. one set to failure with 80% of 1RM loading with a 20 s interset rest interval until a total of 18 repetitions was performed). Total mean time for completing a training session was 57 min for the traditional training group and 35 min for the rest-pause group. After six weeks, strength gains were similar between groups, but the rest-pause group achieved greater gains in hypertrophy in the thigh muscles (rest-pause: 11 ± 14% vs. traditional sets: 1 ± 7%). However, the authors noted that the traditional training group performed 6 repetitions, but training to muscular failure with 80% of 1RM loading would have corresponded to approximately 8–12 repetitions. Thus, the difference in hypertrophy may be due to the higher degree of effort expended in the rest-pause group.

The level of evidence for the rest-pause method remains equivocal, and more research is needed to draw firm conclusions as to its effects on muscular adaptations. Still, when time is a barrier to training, the rest-pause method appears to be an efficient method for improving both strength and especially hypertrophy. It should though be mentioned that the rest-pause method of training is very intense, and some training experience is probably required to train this way in a safe manner, especially when performing complex multi-joint, free-weight exercises.

## Maintenance—How Much Training is Needed to Maintain Strength and Muscle Mass?

For most people, life gets in the way of training at some point (e.g. due to work, family obligations, etc.) and it may be tempting to abandon training completely during these periods. When training is discontinued, muscular gains are preserved for a relatively short period of time (i.e. < 3 weeks), but prolonged periods of detraining ultimately result in both strength loss and atrophy [87, 88]. However, it can be motivating to know that strength and muscle mass appears to be maintained by even small doses of training. In a study by Graves et al. [89], 52 men and women engaged in leg-extension training two or three times per week for 10 weeks. After 10 weeks, there was a mean relative increase in dynamic training load and isometric knee extension strength of 50% and 21% respectively. Next, participants training three times per week were randomized to reduce training levels to two or one time per week, and those initially training twice per week were randomized to training one or zero times per week. After 12 weeks, the group that had refrained from training decreased their isometric knee extension strength by 68% while the groups reducing to two and one time per week had maintained or even slightly increased both their isometric knee extension strength and their training weight. In another study, Bickel et al. asked 70 young (20–35 years) or old (60–75 years) males to perform three sets of three different exercises for the legs, with training carried out three times per week [90]. After 16 weeks, subjects demonstrated considerable increases in both strength and hypertrophy. The subjects were then split into three groups and continued training for 32 weeks with the first group performing no strength training at all, the second group training once every week (three sets of all exercises), and the third group training once every week (one set for all exercises). Participants in both maintenance protocols maintained (or slightly increased) their 1 RM strength during the 32 week period. However, only the young individuals maintained their hypertrophic gains throughout the maintenance period, while the hypertrophic gains acquired by the older individuals returned to baseline levels in both maintenance protocols. This indicates that younger adults can probably maintain muscle mass and strength by training with as little as one brief session per week, while older adults probably need somewhat more weekly volume. These findings correspond with findings in other studies showing that one training session of 3–4 sets for each exercise may be sufficient to maintain muscular strength, at least for some period of time [91, 92]. It should be noted that the studies investigating maintenance lasted only up to 32 weeks; whether strength and hypertrophy can be maintained for an even longer period of a very low training volume is currently not known. Also, the ability to maintain strength and hypertrophy probably differs between individuals and some people could probably manage with even less training, while others may have to train more.

## Warm-up and Stretching—Is It Necessary?

### Warm-up

A warm-up is often recommended at the start of a training session [70]. The warm-up is intended to prepare the body both physiologically and psychologically for training, in the belief, this will enhance performance and reduce the risk of injury [93]. Warm-ups fall into two categories: (a) a general warm-up intended to increase the muscles` and the body’s core temperature (e.g. 5–15 min of low impact exercise such as light-moderate intensity stationary biking), and (b) a specific warm-up intended to increase muscular activation and provide neuromuscular rehearsal of the exercise to be performed (e.g. performing squats with light weights before progressing to heavier squats) [70, 94, 95]. Although a general warm-up is often employed, evidence is limited as to its contribution to strength training. It has been suggested that a combination of general and specific warm-up can enhance 1RM performance [96]. However, most strength training sessions are conducted using submaximal loads. Ribeiro et al. found that neither a general (10 min on an ergometer bike) nor specific warm-up (10 repetitions with 50% of the test loading) provided any benefits regarding fatigue or total repetitions for exercises such as bench press, squats, and arm curl during submaximal strength training (3 sets of 80% of 1RM to failure), compared to no warm-up in young, recreationally-trained men [94]. Thus, from a repetition performance standpoint, a warm-up appears to have limited benefit. Another recent study found that an exercise-specific warm-up resulted in a greater enhancement in peak power output for 1 RM in the high pull compared to a general warm-up [93]. In fact, the general warm-up resulted in only trivial improvements in peak power output compared to no warm-up, and there were no additional benefits obtained from combining both specific and general warm-ups compared to specific warm-up only. Thus, for short duration, power-related performances, a specific warm-up may be sufficient preparation. Support for this hypothesis can be found in a systematic review by McCrary et al., who concluded that strong evidence exists for the use of dynamic warm-ups (performed with greater than 20% of maximal effort) to enhance strength and power in upper-body exercises [97]. The authors further noted that they were unable to find any literature on the effects of warm-up for injury prevention. However, there is some evidence suggesting that specific warm-ups can have a beneficial effect on strength and power, and we would therefore recommend including a specific warm-up for each exercise when time is of the essence. Finally, it is likely that the need for a warm-up is more important when training in the low repetition range using heavy weights, as the initial repetitions could be considered a specific warm-up when training with higher repetitions. There is little support for including general warm-ups when time is of the essence, but some specific warm-ups can be useful, particularly for heavy loads (> 80% 1 RM).

### Stretching

Regular stretching is effective for increasing joint-mobility [8], but it is also frequently promoted by trainers and in the media as an integral part of any training session to improve performance, prevent injuries and reduce delayed onset muscle soreness. However, the scientific evidence does not promote stretching either for improving performance or for reducing injuries and delayed onset muscle soreness. In fact, it has been established that static stretching leads to an acute loss of strength and power, so-called stretch-induced strength loss [8, 98–100], and should therefore probably not be performed before strength training. More specifically, 30–60 min of stretching has been found to cause a 22% (range 14–28%) acute strength loss, while shorter durations of static stretching result in an approximately 8% (range 2–19%) strength loss [98]. Moreover, recent research indicates that regular static stretching may impair chronic measures of strength and power [101]. However, the impairment in strength and power primarily applies to longer sessions and not to short bouts (< 60 s per muscle group) of static stretching [102]. It should be mentioned that dynamic stretching does not appear to reduce strength.

Regarding delayed onset muscle soreness, a 2011 Cochrane review concluded that stretching does not reduce soreness in healthy adults, regardless of whether the stretching is performed before or after the training bout [103]. This finding was supported in a 2018 review that concluded active cool-down after exercise, including stretching, neither appeared to increase recovery nor reduce delayed onset muscle soreness, and likely does not reduce the risk of long-term injury [104]. It also should be noted that resistance training functions as an active form of flexibility training, with evidence indicating similar increases in range of motion when compared to performing a static stretching protocol [105]. Thus, with respect to time-efficiency, stretching should not be prioritized unless an important goal of the training is to increase mobility.

## Practical Applications

Gains in muscle mass can be achieved through a wide spectrum of intensities (loadings), but if low loads (> 15 repetitions) are utilized, training should be performed at or close to muscular failure. This could be particularly relevant in situations where conventional training equipment is not easily available, such as home-based training. However, heavy load and low repetitions are more effective for improving maximal strength capacity, and when heavy loads are employed training to failure appears to be less important. The practical meaningfulness of the strength-related differences between conditions remains questionable; for the general population, the strength gains achieved with low-loads may be sufficient to carry out required activities of daily living.

Each muscle group should be trained with at least four sets per week, and preferably more if additional muscle mass is desired and the necessary additional time can be expended (≥ 10 sets). Considering that less training is needed to maintain, as opposed to gain, strength and hypertrophy, a feasible option could be a form of periodization based on available time, whereby blocks of higher training volume (for increasing strength and hypertrophy) are followed by blocks of lower volume training (primarily for maintenance).

Regarding rest intervals, untrained individuals can flourish with resting 1–2 min between sets while trained individuals probably require ≥ 2 min to maximize muscular gains. The shorter rest intervals should be used when performing exercises for small muscle groups while longer breaks are advised when performing more demanding exercises such as heavy multiple multiple-joint exercises with free-weights.

It is noteworthy that weekly training volume appears to be a more crucial factor than training frequency. While general guidelines recommend training a given muscle group two to three times per week [1], recent reviews and meta-analyses indicate that training frequency appears to be of limited importance when weekly training volume is matched [11]. This is of practical relevance as it allows individuals to choose a weekly training frequency based on their schedule. For instance, some people may have time for several short training sessions spread throughout the week, while others may need to perform a single weekly training session with a longer duration. Alternatively, one can choose a strategy somewhere in between.

While training volume can be quantified in several ways, we recommend defining it as the number of sets performed (close) to failure provided training is carried out within a 6–20 repetition range [106]. Utilizing drop-sets, rest-pause training, and supersets can also be viable methods for increasing training volume and hence stimulating hypertrophy while minimizing training time. Compared to training with traditional sets, drop-sets, rest-pause training, and superset training can induce higher levels of fatigue. Thus, from a practical standpoint, we advise inexperienced lifters to opt for exercise machines over free-weights when performing these advanced time-saving methods with multi-joint exercises as this could be safer and less demanding.

Regarding training equipment, time-efficiency arguments can be made for both machines and free-weights. Machines are arguably more time-efficient for inexperienced lifters as the fixed movement pattern requires fewer coordination/technique skills—allowing people to focus on effort more than form, and changing the resistance is quick and easy. On the other hand, performing different exercises usually requires shifting between machines, which in crowded fitness centers can mean waiting for the machines to become available. Conversely, free-weights provide the ability to perform multiple exercises with the same equipment, which can reduce time spent waiting for equipment. As they are versatile and the space requirements are relatively low, free-weights are also preferable when investing in home-gyms. The choice between free-weights and machines ultimately comes down to preference, availability of equipment, training experience, and training goal; importantly, one does not necessarily have to choose one modality over the other.

Bodyweight- and elastic band training also provide time-efficient options but establishing the appropriate training intensity and maintaining progression over time, as well as achieving high muscle activation in muscles in the lower body, are more challenging with these modalities. It is, however, possible to modify bodyweight exercises to maintain progressive overload over time. For instance, Kotarsky et al. proposed a 10-level progression model for the push-up exercise, starting with wall-push-ups and ending with one-arm push-ups [57]. Still, progressing from one level to the next can be challenging, such as from half push-ups (level 4; e.g., medicine ball under hips) to regular push-ups (level 5). Thus, we recommend performing at least some of the training with free-weights and/or machines and to use bodyweight- and elastic band training as a supplement (e.g. performing one weekly session at a gym using conventional equipment and one home-based session using bodyweight and/or elastic bands). In periods when conventional equipment is not available (e.g. during traveling), bodyweight and elastic band training are viable options. To help quantify the intensity when performing bodyweight- or elastic band training, we advise using a rating of perceived exertion scale such as the BORG-CR10 [48], or the repetitions in reserve scale [107].

When programming strength training for time-efficiency it appears important to primarily focus on bilateral, multi-joint exercises that include the full extent of dynamic movements (i.e. both eccentric and concentric muscle actions). We also advise keeping warm-ups restricted to those that are exercise-specific; stretching should not be prioritized unless a primary goal of training is to increase flexibility. A potential solution for people with both strength- and mobility-oriented goals could be to adopt a superset approach, where strength- and stretching exercises of different muscle groups are performed successively in an alternating manner.

While the focus of this review has primarily focused on alterations in acute training variables, we emphasize that to maximize the effects of training over time, programs should adhere to the principles of specificity and progressive overload [1]. These principles are arguably even more crucial when designing a time-efficient training program as limited training availability requires optimization of all training variables, including how the program is structured over time. For a summary of the practical applications, please see Table 1.

**Table 1:** Summary of practical applications for time-efficient strength- and hypertrophy programs

**How much and heavy should you train﻿?**
Perform ≥ 4 weekly set per muscle group
Increase volume when possible (up to 10 + weekly sets), depending on time constraints
Use 6–15 RM load for strength and hypertrophy
Lighter loads (15–40 RM) can be used if training is performed close to failure (very relevant for home-based training)
**What should you train?**
Perform at least one lower body exercise, and one pulling-, and one pushing exercise for the upper body (preferably bilateral, multi-joint exercises)
E.g. leg press, seated row, and bench press
Use machines and/or free weights based on training goals, availability and personal preference
Elastic bands and bodyweight are viable for home-based training
**Time saving training strategies: Drop-sets, rest-pause training and supersets**
Roughly halves training time compared to traditional trainin﻿g
Primarily beneficial for hypertrophy
Due to safety concerns, we do not advise these methods for heavy compound, free-weight exercises such as the squat and bench press
**Warm-up and stretching**
Stretching should only be prioritized if an important goal is to increase mobility as resistance training in itself promotes improvements in this outcome
General warm-up should not be prioritized when time is of an essence
Specific warm-up can be useful when training with heavy loads (> 80% of 1RM)

## Conclusion

In this narrative review, we have provided an overview of how acute training variables can be manipulated, and how specific training techniques can be used to optimize the training response: time ratio. This knowledge is important for people with limited time for training and for fitness and health professionals. Those with limited time for training should aim to train with ≥ 4 weekly sets per muscle group using a 6–15 RM loading range; if training is performed to volitional failure, a 15–40 repetitions range can also be employed. By performing bilateral, multi-joint exercises, all major muscle-groups can be targeted with as few as three exercises (i.e. a leg pressing exercise, an upper-body pushing exercise, and an upper-body pulling exercise: e.g. leg press, bench press and seated rows). Training can be performed in one, or several shorter sessions—whatever suits the individual. Additionally, advanced training techniques such as drop-sets, rest-pause training and supersets can be used to increase training volume in a more time-efficient fashion. To further reduce training time, individuals could abstain from stretching and a general warm-up, and limit the specific warm-up to the first exercise for each muscle group.

## ﻿Declarations

### Data availability statement

Data sharing is not applicable to this article as no datasets were generated or analysed during the current review.
